# Under Review

**DOI:** 10.1371/journal.pbio.0040033

**Published:** 2006-01-17

**Authors:** Hemai Parthasarathy

## Abstract

Subject to permission from our reviewers, *PLoS Biology* will transfer reviews to any interested journal upon request by the authors.

Why do reviewers do it? Why do they, in a best-case scenario, spend several hours reading, evaluating, and constructively commenting on a manuscript from a group of authors they might not even know? The reasons are many, oft repeated, and about as varied as the comments that reviewers provide on a single paper, on a scale as broad as the human nature from which they derive. Among the more frequently cited motivations are civic duty (good scientific citizenship), loyalty to a particular journal, probable need to read and critique the paper anyway, once it's published, and a desire to control and influence the presentation of science, for noble or ignoble reasons.

Increasingly, however, one hears that the system is overloaded. The best reviewers—the ones who can provide perspective on a field, attention to detail, constructive feedback, and timely responses—can receive as many as ten requests per week. Inevitably, scientists are forced to make choices about which papers to review, and often are willing to review a paper within only a fairly narrow scope that is of immediate relevance to their research. This limits the pool of qualified reviewers for particular papers and hampers the review of interdisciplinary papers. Therefore, if a paper has been reviewed and rejected from one journal, chances are that an editor from a different journal will unwittingly ask some of the same scientific reviewers to review it again. And these reviewers will either agree to review it again, on the basis that they've already seen the paper and can offer a relatively quick assessment, or insist that the authors are provided with the opportunity of a new opinion.

A fresh eye can be an attractive thing, and since no single editorial process can be entirely free from bias and a certain level of incestuousness, a diversified portfolio of journals and editorial models can stand science in good stead, ensuring that the widest possible definition of worthy science is available for postpublication scrutiny. A fresh opinion can also help journal editors make decisions on a paper that might otherwise suffer the ill effects of reviewer fatigue. However, new reviews do not always serve the best interest of the authors. Quite often, reviewers have meted out fair criticisms for the standards or scope of one particular journal, judging the article technically sound but, for example, insufficiently novel. If the door is then closed to resubmission to that journal, an author is sent back to the starting post, when what he or she would prefer is to continue the process to a successful publication rather than have to face new concerns of a second (or third) set of reviewers. Since for virtually any paper, the number of possible concerns is at least equal to (and likely many-fold higher than) the number of possible reviewers, the need to start over can waste an enormous amount of time and energy on the part of authors and reviewers, delay publication, and ultimately reduce the pace of scientific discovery. [Fig pbio-0040033-g001]

**Figure pbio-0040033-g001:**
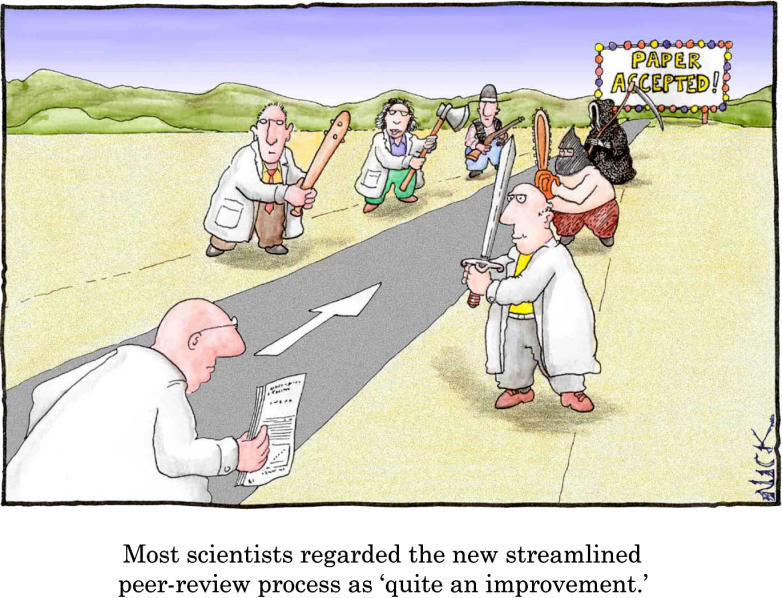
Image: Nick D. Kim

What can be done? Certainly, the answer is not to do away with peer review, which, despite its deficiencies, is frequently identified in surveys as one of the most important features of the traditional process of scholarly publishing (see, for example, the recent report from CIBER's “New Journal Publishing Models: An International Survey of Senior Researchers” [http://www.slais.ucl.ac.uk/papers/dni-20050925.pdf]). Nevertheless, it behooves us all to explore ways to make the process more efficient and more effective. Publishers such as the British Medical Association have explored the benefits of more open peer review, while other journals, such as *Atmospheric Chemistry and Physics* (http://www.copernicus.org/EGU/acp/), have combined closed and open review in a single process.

Publishers that offer a portfolio of journals have come up with another approach to the challenge created by rejection and resubmission; examples include the Nature Publishing Group journals and the Cell Press journals, which allow reviews to be passed from one journal to another within the family. PLoS also provides the possibility of transferring reviews between *PLoS Biology*, *PLoS Medicine*, and community journals (*PLoS Computational Biology*, *PLoS Genetics*, and *PLoS Pathogens*).

However, PLoS goes one step further. Should an author of a paper rejected from *PLoS Biology* wish to have it considered by a journal outside of the PLoS family, we will seek permission from our reviewers to release their identities and confidential reviews to that journal's editors. We hope, of course, that these authors will choose another open-access journal or one of the increasing number of journals with an open-access option. Our respect for our reviewers' confidentiality and for their time is paramount, but within those parameters, we feel that such a service can only benefit the scientific community as a whole. Thus far, we have transferred reviews to *Proceedings of the National Academy of Sciences*, *Development*, and *Conservation Biology*.

Some journals have, in turn, agreed to a similar sharing of reviews with PLoS. Others have not, and their reasons are unclear. For some, financial motives clearly command—the service of procuring reviews is not one to be given away lightly to a competing publisher. But we believe that our first responsibility is to facilitate the communication of scientific research in any way we can. Improving the efficiency of the review process at a time when reviewers are increasingly burdened—and without constraining authors' choices of alternative journals—is just one way we, and all journals, can help.

